# Association of *Alpha B-Crystallin* Genotypes with Oral Cancer Susceptibility, Survival, and Recurrence in Taiwan

**DOI:** 10.1371/journal.pone.0016374

**Published:** 2011-09-07

**Authors:** Da-Tian Bau, Chia-Wen Tsai, Cheng-Chieh Lin, Ru-Yin Tsai, Ming-Hsui Tsai

**Affiliations:** 1 Terry Fox Cancer Research Laboratory, China Medical University Hospital, Taichung, Taiwan; 2 Graduate Institute of Basic Medical Science, China Medical University, Taichung, Taiwan; 3 Graduate Institute of Clinical Medical Science, China Medical University, Taichung, Taiwan; 4 Department of Family Medicine, China Medical University Hospital, Taichung, Taiwan; 5 Department of Otolaryngology, China Medical University Hospital, Taichung, Taiwan; University of Southern California, United States of America

## Abstract

**Background:**

Alpha B-crystallin (CRYAB) is a protein that functions as “molecular chaperone” in preserving intracellular architecture and cell membrane. Also, CRYAB is highly antiapoptotic. Abnormal CRYAB expression is a prognostic biomarker for oral cancer, while its genomic variations and the association with carcinogenesis have never been studied.

**Methodology/Finding:**

Therefore, we hypothesized that *CRYAB* single nucleotide polymorphisms may be associated with oral cancer risk. In this hospital-based study, the association of *CRYAB* A-1215G (rs2228387), C-802G (rs14133) and intron2 (rs2070894) polymorphisms with oral cancer in a Taiwan population was investigated. In total, 496 oral cancer patients and 992 age- and gender-matched healthy controls were genotyped and analyzed. A significantly different frequency distribution was found in *CRYAB* C-802G genotypes, but not in A-1215G and intron2 genotypes, between the oral cancer and control groups. The *CRYAB* C-802G G allele conferred an increased risk of oral cancer (*P* = 1.49×10^−5^). Patients carrying CG/GG at *CRYAB* C-802G were of lower 5-year survival and higher recurrence rate than those of CC (*P*<0.05).

**Conclusion/Significance:**

Our results provide the first evidence that the G allele of *CRYAB* C-802G is correlated with oral cancer risk and this polymorphism may be a useful marker for oral cancer recurrence and survival prediction for clinical reference.

## Introduction

Oral cancer, which is a leading cause of death and disfigurement around the world [1]–[4], has ranked on the 4^th^ cancer in Taiwanese male population [5]. There is an urgent need to develop routine preoperative markers to spare patients with poor prognosis after surgery or other treatment and on the other hand, identify patients at risk of early recurrence and justify prophylactic neck dissection and adjuvant concurrent chemoradiotherapy as well as those who could benefit from various treatments regardless of their tumor size or staging. Those who are identified at higher risk of oral cancer recurrence and/or metastasis should be detected earlier and followed up more frequently to enjoy longer life with the development of useful markers for prognosis prediction.

Alpha B-crystallin (CRYAB) is a member of the small heat shock protein (sHSP) family and a molecular chaperone expressed in various tissues [6], [7]. Recent evidence has established that CRYAB presents not only in eye, but also in heart, skin, brain, spinal cord, and lung tissues [6], [8]. In mammals, there are three classes of crystallins: alpha, beta, and gamma, each contributing equally to the total mass of the lens. From the proteomics or protein level studies, it has recently been recognized that CRYAB may have a role in cancer development. In 2005, it is reported that *CRYAB* was down-regulated at mRNA level from oral cancer patients compared with normal oral mucosa [9]. However, in contrast to the highly expression in normal oral mucosa, patients with negatively or lower CRYAB detected in their tumor sites had better disease-free survival rates than those patients whose tumors stained strongly. On the contrary, it was reported that from a proteomics screening in Taiwan, CRYAB was significantly up-regulated in the primary tissue from oral cancer patients [10]. In 2010, similar results were reported in a mice oral cancer model via concomitantly 8-week treatment with 4-NQO (200 µg/mL) and arecoline (500 µg/mL) [11]. Despite of the disagreements raised among different ethics and populations investigated, the genomic status of *CRYAB* and the linkage between its genotype and clinical outcome are largely unknown.

In order to understand the genomic role of *CRYAB* in oral cancer, we have chosen three single nucleotide polymorphisms (SNPs) of *CRYAB*, A-1215G (rs2228387), C-802G (rs14133), intron 2 (rs2070894), and investigated their genotypic distribution in a large Taiwanese oral cancer population. In addition, the two clinical outcomes contribute to the highest death rate of oral cancer, metastasis and recurrence, were analyzed of their associations with *CRYAB* genotypes.

## Results

**Figure 1:**
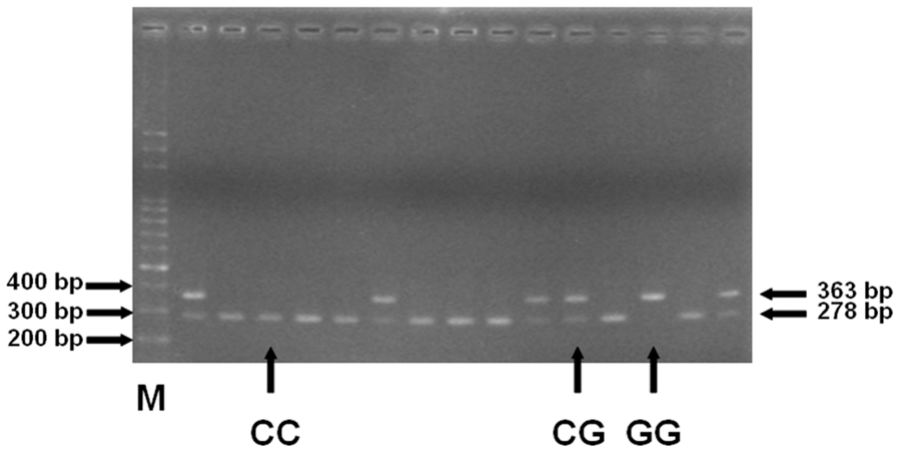
PCR-based restriction analysis of the *CRYAB* C-802G rs14133 polymorphism shown on 2.5% agarose electrophoresis. M: 100 bp DNA size marker, G/G: enzyme indigestible homozygote, C/G: heterozygote, and C/C: enzyme digestible homozygote.

The clinical characteristics of the oral cancer patients and controls are shown in Table 1. There were no significant difference between both groups in their age and sex, while the patients are much higher exposed to the environmental risky factors for oral cancer in Taiwan, smoking, alcohol drinking and betel quid chewing habits (Table 1). The frequencies of the genotypes and alleles for the *CRYAB* A-1215G, C-802G, and intron 2 for the participants are shown in Table 2. Genotype distribution of various genetic polymorphisms of *CRYAB* C-802G is significantly different between oral cancer and control groups (*P*<0.05), while those for A-1215G or intron 2 were not significant (*P*>0.05) (Table 2). Also, the allele distributions of the *CRYAB* C-802G (*P* = 1.49*10^−5^, OR = 1.51, 95%CI = 1.25–1.83), not those of A-1215G (*P* = 0.8593, OR = 0.91, 95%CI = 0.31–2.62) or intron2 (*P* = 0.1366, OR = 1.16, 95%CI = 0.95–1.41), is found to be associated with the susceptibility for oral cancer (Table 2). To sum up, the G allele and GG or CG genotype of *CRYAB* C-802G are associated with oral cancer risk and may be biomarkers for oral cancer detection. The representative PCR-based restriction analyses for the *CRYAB* C-802G polymorphisms are shown in Figure 1.

**Table 1:** Characteristics of oral cancer patients and controls.

Characteristics	Controls (n = 992)			Patients (n = 496)			*P* a
	n	%	Mean (SD)	n	%	Mean (SD)	
Age (y)			66.1 (9.7)			63.8 (8.4)	0.73
Gender							
Male	914	92.1%		469	94.6%		
Female	78	7.9%		27	5.4%		0.16
Indulgence							
Cigarette smokers	526	53.0%		356	71.8%		<0.0001
Areca chewers	506	51.0%		332	66.9%		<0.0001
Alcohol drinkers	445	44.9%		299	60.3%		<0.0001
Histology							
Tongue				247	49.8%		
Buccal mucosa				141	28.4%		
Mouth floor				29	5.8%		
Retromolar trigone				20	4.0%		
Alveolar ridge				13	2.6%		
Palate				12	2.4%		
Lip				11	2.2%		
Others				23	4.6%		

a *P* based on Chi-square test.

**Table 2:** Distribution of *CRYAB* genotypes and allelic frequencies among oral cancer patients and controls.

Polymorphism	Controls	%	Patients	%	*P* a	OR (95% CI)^b^
A-1215G (rs2228387)						
Genotype						
GG	981	98.9%	491	99.0%		1.00 (reference)
AG	11	1.1%	5	1.0%		0.91 (0.31–2.63)
AA	0	0%	0	0%	0.9999	
Allele						
G	1973	99.4%	987	99.5%		
A	11	0.6%	5	0.5%	0.8593	0.91 (0.31–2.62)
C-802G (rs14133)						
Genotype						
CC	703	70.9%	301	60.7%		1.00 (reference)
CG	245	24.7%	158	31.9%		**1.51 (1.18**–**1.92)**
GG	44	4.4%	37	7.4%	**0.0002**	**1.96 (1.24**–**3.10)**
Allele						
C	1651	83.2%	760	76.6%		1.00 (reference)
G	333	16.8%	232	23.4%	1.49*10^−5^	**1.51 (1.25**–**1.83)**
Intron2 (rs2070894)						
Genotype						
CC	688	69.4%	325	65.5%		1.00 (reference)
CT	268	27.0%	150	30.3%		1.18 (0.93–1.51)
TT	36	3.6%	21	4.2%	0.3241	1.23 (0.71–2.15)
Allele						
C	1644	82.9%	800	80.6%		1.00 (reference)
T	340	17.1%	192	19.4%	0.1366	1.16 (0.95–1.41)

a *P* based on two-sided Chi-square test without Yate's correction.

b OR: odds ratio, CI: confidence interval.

**Figure 2:**
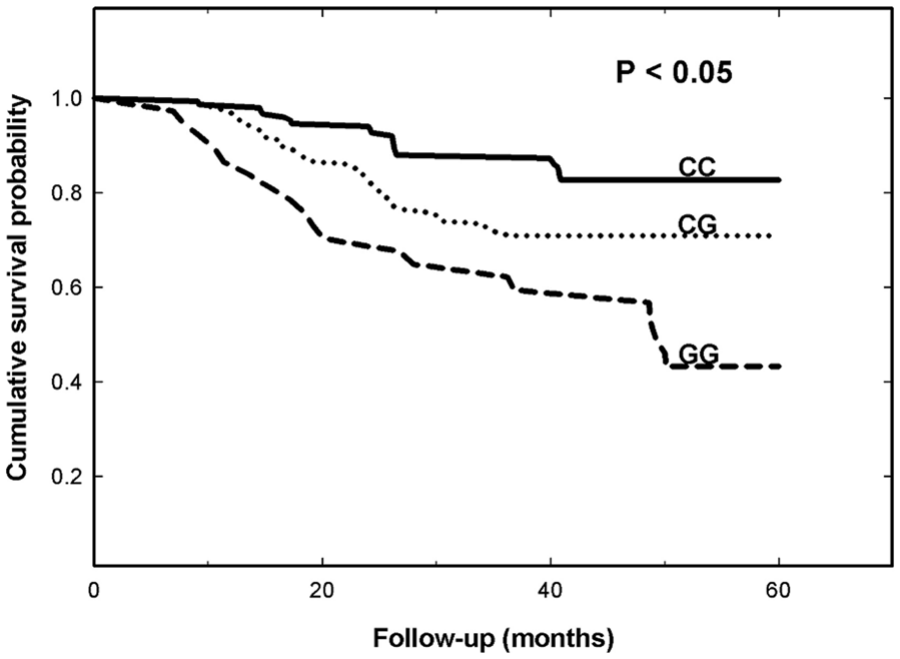
Disease-free survival of oral cancer patients after diagnosis stratified by genotypes of *CRYAB* C-802G. Statistical analysis was performed by the log-rank test.

To evaluate the prognostic value of *CRYAB* genotypes, the relationships among disease-free survival, recurrence, metastasis and *CRYAB* C-802G genotypes were analyzed. First, the oral cancer patients carrying the *CRYAB* C-802G CG had a significant trend toward decreased disease-free survival, and the patients carrying *CRYAB* C-802G GG had the shortest disease-free survival period (Figure 2). The short disease-free may mainly reflect local recurrence. More than 80% (30 of 37) of the patients carrying *CRYAB* C-802G GG had nodal recurrence without an advanced N stage (N0-1) at the first diagnosis. Interestingly, the patients would have frequent recurrence and high second primary tumor rates within the following five years. Second, compared with those with CC genotype, the patients carrying *CRYAB* C-802G CG or GG genotype had a higher recurrence rate within the following five years (*P* = 0.228, OR = 2.08, 95%CI = 1.11–3.92), but not a higher metastasis rate (Table 3).

**Table 3:** Association of *CRYAB* C-802G genotype with oral cancer recurrence and metastasis.

Patient Status	*CRYAB* C-802G rs14133			
	CC	CG+GG	*P* a	OR (95% CI) b
Recurrence Status				
No recurrence >5 years	282	171		1.00
Recurrence <5 years	19	24	**0.0228** c	2.08 (1.11–3.92)
Metastasis Status				
No metastasis >5 years	279	178		1.00
Metastasis <5 years	22	17	0.6101	1.21 (0.63–2.34)

a *P* based on two-sided Chi-square test without Yate's correction.

b The ORs were estimated with multivariate logistic regression analysis.

c Statistically identified as significant.

## Discussion

The study aimed to investigate the association of *CRYAB* genotypes and clinicopathopogical variations in Taiwan oral cancer patients. It has recently been recognized that CRYAB protein may play a role in oral cancer development. In previous literature, it was reported that CRYAB was significantly over expressed in the primary tissue from oral cancer patients in Taiwan [10]. In 2010, in a tongue cancer mice model performed by concomitantly 8-week treatment with 4-NQO (200 µg/mL) and arecoline (500 µg/mL) and withdraw in the following 20 weeks, the cells of the tumor sites had higher expression of CRYAB than the counterpart cells of the sham-treated mice [11]. However, there were also some findings challenging this up-regulation association [9], [12]. This may be due to that different populations of different ethics, genetic background, cultures, and environment exposure were investigated. From the viewpoints of cell-line based studies, it was demonstrated that overexpression of CRYAB in transformed immortalized human mammary epithelial cells demonstrated neoplastic features and luminal growth and these changes were inhibited when CRYAB expression was silenced using RNA interference [13]. Overexpression of CRYAB in human mammary epithelial cells also formed invasive mammary carcinomas in nude mice, induced epidermal growth factor and anchorage independent growth, increased cell migration and invasion, and activated the mitogen-activated protein kinase/extracellular signal-regulated kinase (MEK/ERK) pathway, suggesting that CRYAB could be considered an oncoprotein [14]. However, there is not yet any study performed from the DNA level to investigate the important role of *CRYAB* in carcinogenesis.

Based on the previous differential expression evidence, we were strongly interested and chose the three SNPs of *CRYAB*, two at the promoter region (A-1215G and C-802G) and one at the intron 2 (intron2), to investigate their associations with oral cancer risk and prognosis. We found that *CRYAB* C-802G polymorphism, not A-1215G or intron2, was associated with increased risk of oral cancer (Table 2), and the local recurrence rate (Table 3). Also, the oral cancer patients carrying GG or CG at the polymorphic site had lower 5-year survival rate than those carrying homologous CC (Figure 2). Interestingly, the patients carrying GG at *CRYAB* C-802G were recorded to have much more frequent recurrence and second primary rates. This may indicate that *CRYAB* C-802G could be a predicator for oral cancer progression direction. Possibly the genetic polymorphism directly affects the differential patterns of the CRYAB protein, at the expression and/or functional levels, and indirectly imbalances the normal functions of other CRYAB-related genes and proteins, which may result in the oral carcinogenesis. At the same time, the alteration of CRYAB protein expression in the extracellular matrix may cause the subtle changes of the microenvironment near the primary oral tumor, for the recurrence, but not for the metastasis. This can be justified by the role of CRYAB in the tyrosine kinase signaling, that could be easily altered in cancer cells. The reduced expression of CRYAB has been firstly reported to be associated with a negative prognosis in 2003 [15].

Approximately 10% of early-stage head and neck squamous cell carcinoma patients develop locoregional recurrence and 15% to 25% develop second primary tumors within 5 years of initial diagnosis [16], [17]. As diagnostic and therapeutic approaches continue to develop, the ability to accurately predict second primary tumor/recurrence in early-stage oral cancer patients would facilitate intensive surveillance or targeted interventions for high-risk patients and thereby reduce mortality and morbidity. In this study, the patients carrying *CRYAB* C-802G CG or GG genotype were found to have a higher recurrence rate within the following five years, but not a higher metastasis rate (Table 3).

The occurrence of second primary tumors may be due to the subtle alterations of the microenvironment which have been accumulated to reach the threshold of tumorigenesis in the patients of risky genotypes, such as GG at *CRYAB* C-802G. The functional study of this SNP and how the CRYAB protein interacts with proteins in extracellular matrix in oral carcinogenesis also need further investigations. In the future, collective evidence from larger and different cohorts using this SNP may help to oral cancer staging, outcome direction prediction, and more effective and integrative strategy.

It is firstly found that the SNP at the promoter region of *CRYAB,* C-802G, is associated with oral cancer susceptibility, recurrence, and 5-year disease-free survival, but not metastasis. Since poor local-regional control and easy recurrence are the main causes of treatment failures in oral cancer therapy, the results of this study may provide more predictive guidance information for not only the prevention, but the care, therapy and follow-up of those patients at higher risk of cancer recurrence and lower 5-year survival rate.

## Materials and Methods

### Study population and sample collection

Four hundred and ninety six cancer patients diagnosed with oral cancer were recruited at the outpatient clinics of general surgery between 2005–2008 at the China Medical University Hospital, Taichung, Taiwan, Republic of China. The clinical characteristics of patients include histological details were all graded and defined by expert surgeons. All patients voluntarily participated, completed a self-administered questionnaire and provided peripheral blood samples. Double number of non-cancer healthy volunteers as controls were selected by matching for age, gender and some indulgences after initial random sampling from the Health Examination Cohort of the hospital. The exclusion criteria of control group included previous malignancy, metastasized cancer from other or unknown origin, and any familial or genetic diseases. Both groups finished a short questionnaire which included some indulgences and they were recorded. Our study was approved by the Institutional Review Board of the China Medical University Hospital and written-informed consent was obtained from all participants.

### Genotyping assays

Genomic DNA from oral cancer and health control subjects were prepared from peripheral blood leucocytes using a QIAamp Blood Mini Kit (Blossom, Taipei, Taiwan) and further processed according to previous studies [18]–[24]. Briefly, the following primers were used for *CRYAB* A-1215G (rs2228387): 5′-ACCTGTTGGAGTCTGATCTT-3′ and 5′-ATGCACCTCAATCACATCTC-3′; for *CRYAB* C-802G (rs14133): 5′-TTGACCATCACTGCTCTCTT-3′ and 5′-TTGGCAATGTGACACATACC-3′; for *CRYAB* intron 2 (rs2070894): 5′-GTCTAGAAGACTAAGTTAGG-3′ and 5′-AGAGAAGTCACAACTCAAGT-3′; The following cycling conditions were performed: one cycle at 94°C for 5 min; 35 cycles of 94°C for 30 s, 55°C for 30 s, and 72°C for 30 s; and a final extension at 72°C for 10 min. The PCR products were studied after digestion with *Fau I*, *Fat I*, and *DPN I*, restriction enzymes for A-1215G (cut from 212 bp A type into 67+145 bp G type), C-802G (cut from 363 bp G type into 85+278 bp C type), and intron 2 (cut from 363 bp T type into 74+339 bp C type), respectively.

### Statistical analyses

In our study, *o*nly those matches with all SNPs data (case/control  = 496/992) were selected into final analyzing. To ensure that the controls used were representative of the general population and to exclude the possibility of genotyping error, the deviation of the genotype frequencies of *CRYAB* SNPs in the control subjects from those expected under the Hardy-Weinberg equilibrium was assessed using the goodness-of-fit test. Pearson's two-sided Chi-square test or Fisher's exact test (when the expected number in any cell was less than five) was used to compare the distribution of the *CRYAB* genotypes between cases and controls.

The primary outcome was disease-free survival. The endpoints included local cancer recurrence and metastasis. Follow-up information was available for all patients at the 5-year time point. Disease-free survival time was calculated from the date of treatment until the time of recurrence, defined as disease recurrence at the same site or the detection of metastases, including recurrence in the neck lymph nodes. The genotypes were coded assuming an allele dose-effect (CC wild-type = 0, CG heterozygous carrier of the mutated allele = 1, GG homozygous carrier of the mutated allele = 2). Disease-free survival curves were generated by the Kaplan-Meier method and verified by the log-rank test. The significance level was set at *P*<0.05.

## References

[1] Caplan DJ, Hertz-Picciotto I (1998). Racial differences in survival of oral and pharyngeal cancer patients in North Carolina.. J Public Health Dent.

[2] Moore RJ, Doherty DA, Do KA, Chamberlain RM, Khuri FR (2001). Racial disparity in survival of patients with squamous cell carcinoma of the oral cavity and pharynx.. Ethn Health.

[3] Shiboski CH, Shiboski SC, Silverman S (2000). Trends in oral cancer rates in the United States, 1973–1996.. Community Dent Oral Epidemiol.

[4] Swango PA (1996). Cancers of the oral cavity and pharynx in the United States: an epidemiologic overview.. J Public Health Dent.

[5] Department of Health Taiwan (2008). Cancer registration system annual report..

[6] Bhat SP, Nagineni CN (1989). alpha B subunit of lens-specific protein alpha-crystallin is present in other ocular and non-ocular tissues.. Biochem Biophys Res Commun.

[7] Iwaki T, Kume-Iwaki A, Goldman JE (1990). Cellular distribution of alpha B-crystallin in non-lenticular tissues.. J Histochem Cytochem.

[8] Sax CM, Piatigorsky J (1994). Expression of the alpha-crystallin/small heat-shock protein/molecular chaperone genes in the lens and other tissues.. Adv Enzymol Relat Areas Mol Biol.

[9] Chin D, Boyle GM, Williams RM, Ferguson K, Pandeya N (2005). Alpha B-crystallin, a new independent marker for poor prognosis in head and neck cancer.. Laryngoscope.

[10] Lo WY, Tsai MH, Tsai Y, Hua CH, Tsai FJ (2007). Identification of over-expressed proteins in oral squamous cell carcinoma (OSCC) patients by clinical proteomic analysis.. Clin Chim Acta.

[11] Chang NW, Pei RJ, Tseng HC, Yeh KT, Chan HC (2010). Co-treating with arecoline and 4-nitroquinoline 1-oxide to establish a mouse model mimicking oral tumorigenesis.. Chem Biol Interact.

[12] Boslooper K, King-Yin Lam A, Gao J, Weinstein S, Johnson N (2008). The clinicopathological roles of alpha-B-crystallin and p53 expression in patients with head and neck squamous cell carcinoma.. Pathology.

[13] Solares CA, Boyle GM, Brown I, Parsons PG, Panizza B (2010). Reduced alphaB-crystallin staining in perineural invasion of head and neck cutaneous squamous cell carcinoma.. Otolaryngol Head Neck Surg.

[14] Moyano JV, Evans JR, Chen F, Lu M, Werner ME (2006). AlphaB-crystallin is a novel oncoprotein that predicts poor clinical outcome in breast cancer.. J Clin Invest.

[15] Stronach EA, Sellar GC, Blenkiron C, Rabiasz GJ, Taylor KJ (2003). Identification of clinically relevant genes on chromosome 11 in a functional model of ovarian cancer tumor suppression.. Cancer Res.

[16] Khuri FR, Kim ES, Lee JJ, Winn RJ, Benner SE (2001). The impact of smoking status, disease stage, and index tumor site on second primary tumor incidence and tumor recurrence in the head and neck retinoid chemoprevention trial.. Cancer Epidemiol Biomarkers Prev.

[17] Khuri FR, Lee JJ, Lippman SM, Kim ES, Cooper JS (2006). Randomized phase III trial of low-dose isotretinoin for prevention of second primary tumors in stage I and II head and neck cancer patients.. J Natl Cancer Inst.

[18] Bau DT, Tsai MH, Lo YL, Hsu CM, Tsai Y (2007). Association of p53 and p21(CDKN1A/WAF1/CIP1) polymorphisms with oral cancer in Taiwan patients.. Anticancer Res.

[19] Bau DT, Tseng HC, Wang CH, Chiu CF, Hua CH (2008). Oral cancer and genetic polymorphism of DNA double strand break gene Ku70 in Taiwan.. Oral Oncol.

[20] Chiu CF, Tsai MH, Tseng HC, Wang CL, Tsai FJ (2008). A novel single nucleotide polymorphism in ERCC6 gene is associated with oral cancer susceptibility in Taiwanese patients.. Oral Oncol.

[21] Chiu CF, Tsai MH, Tseng HC, Wang CL, Wang CH (2008). A novel single nucleotide polymorphism in XRCC4 gene is associated with oral cancer susceptibility in Taiwanese patients.. Oral Oncol.

[22] Hsu CF, Tseng HC, Chiu CF, Liang SY, Tsai CW (2009). Association between DNA double strand break gene Ku80 polymorphisms and oral cancer susceptibility.. Oral Oncol.

[23] Tsai MH, Tseng HC, Liu CS, Chang CL, Tsai CW (2009). Interaction of Exo1 genotypes and smoking habit in oral cancer in Taiwan.. Oral Oncol.

[24] Tseng HC, Tsai MH, Chiu CF, Wang CH, Chang NW (2008). Association of XRCC4 codon 247 polymorphism with oral cancer susceptibility in Taiwan.. Anticancer Res.

